# Lymphocyte expansion in bioreactors: upgrading adoptive cell therapy

**DOI:** 10.1186/s13036-021-00264-7

**Published:** 2021-04-13

**Authors:** Oscar Fabian Garcia-Aponte, Christoph Herwig, Bence Kozma

**Affiliations:** Research Area Biochemical Engineering, Institute of Chemical, Environmental and Bioscience Engineering, TU Wien, Gumpendorferstraße 1a, 1060 Vienna, Austria

**Keywords:** Adoptive cell therapy, ATMP, Bioreactor, Expansion, Lymphocyte, Rocking motion, Stirred reactor, Perfusion reactor, NK cell, T cell

## Abstract

Bioreactors are essential tools for the development of efficient and high-quality cell therapy products. However, their application is far from full potential, holding several challenges when reconciling the complex biology of the cells to be expanded with the need for a manufacturing process that is able to control cell growth and functionality towards therapy affordability and opportunity. In this review, we discuss and compare current bioreactor technologies by performing a systematic analysis of the published data on automated lymphocyte expansion for adoptive cell therapy. We propose a set of requirements for bioreactor design and identify trends on the applicability of these technologies, highlighting the specific challenges and major advancements for each one of the current approaches of expansion along with the opportunities that lie in process intensification. We conclude on the necessity to develop targeted solutions specially tailored for the specific stimulation, supplementation and micro-environmental needs of lymphocytes’ cultures, and the benefit of applying knowledge-based tools for process control and predictability.

## Background

In the process of understanding cancer, clinical research has developed a resourceful toolbox of treatment options ever increasing in complexity. From surgery and radiation therapy, going through chemotherapy and biologics, we have arrived to the field of Cancer Immunotherapy [1], an approach that merges with the innovative area of Advanced Therapy Medicinal Products (ATMPs) to develop the specialty of Adoptive Cell Therapies (ACT).

This branch of immunotherapy is defined as the intravenous administration of ex vivo expanded immune effector cells that are capable of selective cytotoxicity. It exploits the immune system’s ability to distinguish between pathologic and healthy tissue [2, 3]. ACT has been characterized as a “living” treatment that can be enhanced by means of gene modification because cells continue to function in vivo after they have been infused back into a patient [4]. ,To date, many cells have been used for ACT, including Lymphokine-Activated Killer (LAK) cells, Tumor-Infiltrating Lymphocytes (TILs), Cytotoxic T Lymphocytes (CTLs), Cytokine-Induced Killer (CIK) cells, γδ T cells, Regulatory T (TReg) cells, Natural Killer (NK) cells, engineered T cells (T-Cell Receptor (TCR T) cells and Chimeric Antigen Receptor (CAR) T cells) [2, 5, 6].

Unfortunately, these cells remain as a limited therapeutic option that is only applied to a small number of patients. Partly because of significant knowledge gaps on their clinical effectiveness and cost/benefit ratio and a strong dependency on highly specialized methods, materials and equipment, therefore the number of products approved for commercialization is reduced [7, 8]. As the last decades saw progress in the understanding of lymphocyte biology and different companies are developing high throughput systems for ACT manufacturing [9], it is expected that this field will experience a quick clinical and technical expansion, that requires process intensification and innovative solutions from engineers. Hence, there will be a future push to technologize ACTs, from hospital-oriented to industrially relevant manufacture processes.

**Fig. 1:**
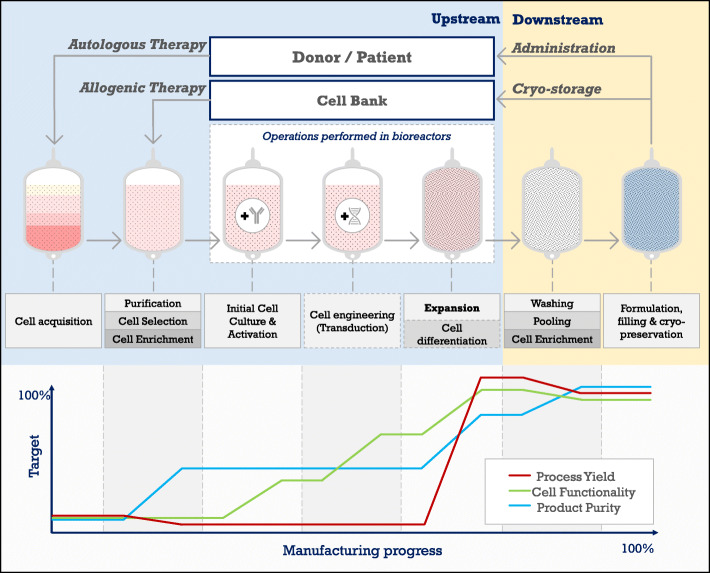
General upstream and downstream steps of a cell therapy product from autologous or allogenic source. The graph shows the contribution of the different unit operations to final cell yield (red), functionality (green) and purity (blue). This review focuses on the expansion process

The manufacturing of an ACT product usually begins with a mixed lymphocyte population from a patient’s biopsy, or from apheresed Peripheral Blood Mononuclear Cells (PBMCs) (Fig. 1). It can also be started by differentiating a cell subset from Hematopoietic Stem Cells (HSC) and lymphoid progenitors generally obtained from Umbilical Cord Blood (UCB). After cell acquisition, several workflows can be followed depending on the intended application. In upstream, most of the protocols include cell selection, enrichment, purification, activation, stimulation, gene modification and expansion, while downstream processes include pooling, further enrichment, formulation and cryopreservation [10–13]. Independently from the workflow, and because ACT doses composed of high cell numbers generally produce more desirable therapeutic outcome [14, 15], the cell expansion process is a common factor in any ACT protocol, being subjected to the greatest research efforts and the most significant body of user experience [16]. Expansion’s ubiquity highlights its importance for ACT’s optimization, relying on the application of Quality by Design (QbD) principles for sound bioprocess understanding. However, optimizing for a process focused only on high cell output could narrow the Critical Quality Attributes (CQAs) down to the productivity issue. In that sense, ACT would not benefit from an integrative clinical view, able to compensate for regulatory and engineering constraints [17] in a broader context that considers yield, cell purity and product functionality.

The aim of this review is to give a comparative overview of lymphocyte expansion in bioreactors, assessing their ability to generate sufficient, functional and correctly differentiated cell populations, with considerations to process flexibility, controllability and scale. We explore the manufacturing of lymphocytes primarily from PBMCs and biopsies, summarizing the outcomes from the diverse expansion processes but taking the comparability issues arising from the wide range of stimulation and supplementation strategies into the picture, apart from the selected bioreactor technology. Lymphocyte manufacturing from stem cells is excluded from this review as it adds an extra layer of complexity to the comparison exercise. We first provide a context on the general culturing requirements for lymphocytes, later discussing the challenges of transitioning to technologized manufacturing. Given that context, a set of requirements for bioreactor design and comparison for allogenic and autologous ACT is presented. We then review and categorize the available bioreactor technologies based on published results on process yield, cell purity and product functionality. Finally, we propose further knowledge intensive approaches that could be useful to take advantage of the data intensive environment that bioreactors bring to the field of ACT.

## The complexity of lymphocyte expansion

Compared to small molecules and biologics, living cells are much more complex: they sense their surroundings, react to their environment and express varied and adjustable behaviors [18]. Furthermore, they have some unique features [19], including the ability to specifically distinguish, bind and kill abnormally growing cells by selectively switching metabolic pathways to enhance the production of cytotoxic substances [20]. Because of this complex biological setting, any small change in the culture environment may result in the alteration of product *quality* [21], a concept that acquires a greater dimension, as it becomes associated with information on cell state, phenotype, functionality and identity [22].

The unpredictable behavior of lymphocytes during culture causes noticeable variations in expansion rates amid manufacturing [23]. This inherent variability hinders any comparison between expansion protocols in order to conclude and organize best practices. At the core of this issue relies donor heterogeneity as differences related to age, gender, health issues or ethnicity are frequent [24]. Donor variability is also linked to process performance and lymphocyte sensitivity to process parameters [25]. Modeling for process predictability, associated with a thorough characterization of raw materials to compensate for source’s variability can improve process understanding, accelerating the establishment of new cellular therapies [14]. To make it even more complex, lymphocytes can tune their communication with the environment by modifying their receptor/ligand repertoire, changing cellular sensitiveness to external substances and surfaces [19]. These aspects often generate an undesired outcome: when subjected to extensive cultivation, cells are prone to develop phenotypic changes (e.g. differentiation, senescence or immunogenicity) or genetic changes (e.g. mutations, gene deletions or chromosomal aberrations) that can severely undermine their safety and efficacy profiles. Therefore, higher yield due to prolonged expansion often correlates with the selection of more proliferative cell subpopulations, which can be less efficient for their designed function [14].

Additionally, immune cells must be stimulated by carefully integrating selection and activation steps during the expansion process. There are several technologies available for the activation of immune cells, including cell-based activation, bead-based activation, and antibody-based activation. Antigen Presenting Cells (APCs), as cell-based activators, are endogenous agents that provide an in vivo-like stimulation but they are expensive to use in a GMP environment, difficult to remove from the final cell population, variable in their potential to induce activation and may be scarce when isolated from donor samples [11]. Traditionally, immune cell expansion has also relied on the supplementation with animal or human serum. However, the use of serum may generate safety risks of infusion and increases process variability due to batch-to-batch differences [11, 26, 27]. Besides antigen-induced activation, stimulation with cytokines is another factor that influences the composition, quality and phenotype of the final cell product. T cells are generally produced by IL-2, IL-7 and/or IL-15 stimulation [28], while most current NK cell expansion protocols include the use of IL-2 and IL-15 [29, 30]. Complex, precisely scheduled cytokine cocktails for culture stimulation can also be used under certain expansion protocols.

Through the usage of these stimulation agents, the expanded cells undergo frequent metabolic changes. They can move into quiescence or active status, start the division cycle, enter apoptosis or differentiate. Knowing what process is triggered in which cells is important, yet most expansion results just consider the overall expansion rate of a given subset of cells. Furthermore, metabolism is not only relevant as a descriptor of cell growth. There is a growing body of evidence that shows immune cell metabolism to be essential to cell functionality. For example, glycolysis and oxidative metabolism have been shown to modulate classical anti-tumor effector functions of NK cells [31]. Thus, positive and negative modulation of certain metabolic triggers could be used to control ex vivo expansion and direct cell functionality. Amino acid modulation is another tool that may enhance cell expansion, because some of them, such as glutamine, arginine and tryptophan, have been found to influence lymphocyte proliferation [32].

Summarizing, lymphocytes could be portrayed as delicate cells requiring very meticulous culturing. Their behavior can be unpredictable to some extent, because of a combination of factors that include donor and cell population heterogeneity, frequent metabolic changes, high sensitivity to culture environment and strong dependency on an accurate stimulation strategy that mimics typical in vivo conditions. This complexity demands an expansion process that is sensitive and flexible enough to compensate for variability. This is offered by various bioreactor systems that were proven to be applied for lymphoid cultures.

## From static cultures to intensified processes

Despite of the tight control needed for efficient ACT manufacturing, immune cells are still frequently expanded in static systems equipped with limited monitoring capacity [10, 19, 23]. These platforms (plates, flasks and bags) depend on incubators and are restricted to a batch-and-split mode which periodically divides and refills the culture with medium to cope with the cells’ metabolic activity and stimulation requirements, therefore these cultures are highly susceptible to contamination as multiple open vessels are needed to create a single product [33]. Furthermore, the medium renewal cycles cause frequent nutrient and metabolite fluctuations that may trigger high phenotypical variability [19]. As a result, ACT cells are still manufactured through processes and methods that have been characterized as “archaic, scarcely controlled and incomparable” [34]. Because of their simplicity, cell therapy companies may initiate clinical trials using static systems, requiring further assessment as key differences in parameters such as shear stress, culture conditions, and cell-to-cell interactions may cause a divergent biological profile as the cells are moved to a bigger scale dynamic set-up [35].

Quality testing, which includes complex functionality assays, should be carried in a timely manner, as ACT products are generally used or preserved briefly after production, increasing the risk of uncertainty and therapeutic mistakes [36]. This implies that Process Analytical Technology (PAT) alone is not able to provide robust information to address most quality questions. Because of that, discrete in-process characterization of cell status during manufacture is generally out of phase with properties continuously monitored using PAT tools, which are inferential in nature (e.g. DO, pH, glucose consumption or cell density) [37]. However, our comprehension of cell status, including metabolomics, clonogenicity and cell cycle regulation is significantly improving [38].

**Fig. 2:**
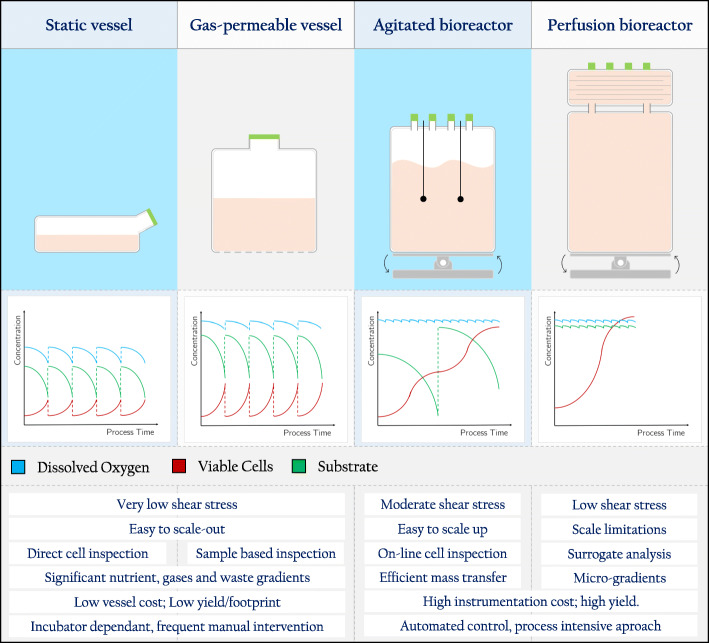
Main characteristics of static and dynamic culture vessels and their influence on process variability. Typical trends of viable cell density (red line), nutrient concentration (green line) and dissolved oxygen (blue line) for each culture vessel type

Most of the bioreactors used for the cultivation of therapeutic cells originate from vessels and technology created for upstreaming bacteria or yeast [14]. However, it is important to note that these systems do not focus on cell integrity and functionality but on maximizing yield, thus requiring refitting to face the challenge of generating a healthy and functional cellular product [38]. Bioreactors allow process scale up with high standardization and reproducibility, while enabling the evaluation of the influence of process parameters on culture performance [39]. In the same way, process intensification through the implementation of mechanistic modeling and PAT tools, along with the use of automated culturing techniques, facilitates to reach better control over cell expansion [14] (Fig. 2).

A bioreactor’s capability to monitor and control critical process parameters is a highly valuable characteristic yet to be optimally explored with lymphocyte cultures. To profit on these abilities, several bioreactor designs were already tested for lymphocyte culturing. These different bioreactor configurations (Fig. 2) are generally suitable for a specific field of ACT (either allogenic or autologous applications). However, as the cultured cells have in principle the same needs, a general set of requirements towards maximizing bioreactor capabilities can be formulated, guiding the transition from static cultures to intensified processes.

## Requirements of bioreactors for lymphocyte culture

Although every cell therapy process has unique elements, it is not practical to design specialized devices for each specific product. Instead, ACT products should be grouped on shared process characteristics, defining strategies and technologies that fit better for each category as a whole [40]. In that regard, ACT can be performed using two general principles: autologous and allogenic. In the autologous setting, a batch is individually produced from a patient’s biopsy, isolating and culturing the cell population of interest. In the allogenic workflow, cell source is a universal donor platform with highly expandable cells that have similar scale requirements as the manufacturing of cell derived proteins and the cell product may target multiple patients [25]. Process-wise, increasing vessel scale and ensuring culture performance (scale-up) is related to allogeneic approaches, while parallelizing several independent units (scale-out) is generally the goal in optimizing autologous therapy [22]. An autologous batch size is not expected to exceed more than a few liters volume, because of the limited amount of starting material and the time sensitivity of the cells to retain their functionality. Thus, scaling up autologous is not useful and scaling out for multiple batches still requires a thorough assessment of technical capacities [35]. This delicate setting for autologous cell therapy drives bioprocess development towards automation [11, 25], as the ideal autologous platform should compensate for the effects of varying culture conditions on CQA’s performance [40]. The allogenic set up, on the other hand, requires appropriate inoculation levels with minimal seed adaptation to maximize the expansion outcome. Therefore, the possibility of having a set of vessels geometrically and dynamically comparable is highly relevant [41]. In the same way, achieving consistent process reproducibility is necessary for a standardized and safe allogenic platform, thus, allogenic bioprocess development is mostly driven towards process control than workflow automation. To harness a bioreactor’s full potential, its design and application should be fitted to the challenges of cultivating lymphocytes and the supplements necessary for their growth. These are, in the view of the authors, and based on previous frameworks of requirements [14, 19, 25, 34, 35, 42], the main standards to be fulfilled by a culturing platform for ACT.

### Suitable vessel size and scalability

Cell-based therapies often require the application of vast quantities of cells (10^8^–10^10^) to patients therefore the space required for their growth is a practical limitation. Assuming a culture density of 10^6^ to 10^7^ cells/mL (a high value for ACT), it would demand a volume starting from few milliliters up to tens of liters during culture [43]. The available bioreactor scale must be flexible enough to fully accommodate the range of cell growth across all feasible batches, and to compensate for the expected potential growth variability from the source [25]. To achieve this, ultra-high cell density cultures or an industrial scale production that is able to maintain uniform culture conditions are required [39]. Some current expansion processes include a preliminary stage where cells are activated and rapidly multiplied in static systems, generating enough cells for bioreactor inoculation. However, enough bioreactor space for the actual expansion is still necessary.

### GMP compliance

To avoid cross contamination (between different batches or patients) and microbiological contamination, closed systems (bags, expansion sets, flasks), incubators and hoods should be used [36]. Bioreactors should guarantee sterility by keeping a closed system [19]. Each manipulation step (e.g. inoculation, activation, transduction, media changes, stimulation, sampling, washing) creates a risk for error and contamination that may lead to a failed run [36]. For that purpose, single-use, closed, disposable cell production “kits” may represent a desired design strategy for patient-specific cell therapy manufacturing protocols [44], particularly if such kits can be designed for simplicity [43].

### Process control

Once the specific requirements for the cells being expanded have been defined, process parameters such as temperature, shear stress, dissolved oxygen (DO) and CO_2_ and environmental variables like osmolality and pH must be kept at optimal values [14]. .Extensive, online process monitoring and integrated control is required for adaptation to process changes [45]. DO and pH of the medium are typically held constant to provide a consistent environment supporting optimal cell expansion. DO and pH signals, are valuable for assessing the status of the expansion medium and cell proliferation, triggering a proportional feeding strategy [41], although this is a fairly limited approach. Some technologies that should be considered for ACT process monitoring and control are included in Table 1. The final goal of process monitoring should be to find descriptors that can give information about the influence of batch-to-batch or donor-to-donor variability on the expansion process [58]. The best approach for process control development would be to use PAT data to facilitate process related decisions in real-time, or even predictively. This can include decision points for transduction, perfusion initiation, harvest point, or even quality control release based on minimum viability or endotoxin level. Ideally, such technologies would evolve to measure surface markers expression of key phenotypic markers.

**Table 1:** Advanced process monitoring tools for ACT

Tool	Application	Type	Ref.
Raman spectroscopy	Metabolite monitoring (glucose, lactate, amino acids).	On-line	[46]
	Total Cell Concentration.	On-line	[46]
	Cell identity determination (phenotype & activation).	At-line	[47, 48]
Sequential injection capillary electrophoresis	Metabolite monitoring (glucose, lactate, amino acids).	At-line	[49]
	Cell concentration.	At-line	[49]
FT-IR spectroscopy	Glucose monitoring.	On-line	[50]
Electrical impedance	Cell-mediated cytotoxicity and cell adhesion.	At-line	[51–53]
Biosensors for acidification measurement	Metabolite monitoring (lactate).	On-line	[54]
Biosensors – optical	Cytokine quantification.	Potential	[55, 56]
Gas chromatography-mass spectrometry	Volatile organic compound (VOC) emissions profiling – metabolic monitoring.	On-line	[57]

### Handling of shear stress

Ex vivo expansion of all immune cell types should avoid mechanical stress by chaotic, inhomogeneous medium dynamics [19]. It has been long established that animal cells are sensitive to shear, which, above certain levels, compromises their viability. Besides the direct effect that mechanical forces can exert on a cell membrane’s integrity, animal cells are adapted to the environment of each tissue, evolving sensitive mechanisms for detecting shear changes. To develop an acceptable understanding of how these forces influence cell behavior, it is necessary to recreate similar level of shear forces than found in the body within a bioreactor, allowing for a detailed characterization and control of the mechanotransduction process [59] and the direct effects of shear on the cells. Importantly, agitation must be designed to manage not only shear exposure of cells, but also the efficiency of mass transfer, suspension of cells and avoidance of heterogeneities that may cause cell inconsistencies [25].

### Representative sampling

The designed bioreactor process should stay out of any artificial deleterious influences on cell integrity by passaging and reseeding the cells, as it may decrease total yield [19]. Sampling and harvesting of cells, medium, or both should be also designed with simplicity in mind. Taking samples has certain drawbacks that need to be mitigated [35]: to get a representative bioreactor sample, a significant volume should be drawn, which can impact on yield, especially if multiple small scale vessels are used for the cell expansion. Repeated sampling can also increase the risk of contaminating the bioreactor. Issues that need to be resolved in such cell therapy process development platforms include deciding on the amount of cells needed to reflect heterogeneity and the usage of live cell-based image analysis and “lab-on-chip” strategies [43].

### Stimulation and supplementation

Media changes in bioreactors are usually done by nutrient addition, or by total or partial media replacement, or by perfusion. If a cell culture produces non-damaging levels of waste products, concentrated levels of nutrients can be added over time to feed the growing culture. Inevitably, waste metabolites such as lactate and ammonia start to accumulate, and either media replacement or perfusion is required. Perfusion, in which fresh media is gradually fed and old media is removed while the cells are retained, is the ideal way to intervene and still maintain a stable environment for cell therapy [35]. It also should be noted that cell exhaustion can be induced by current activation methods, which generally also demand careful operator attention [32]. Because of that, precise optimization of the feeding of nutrients and cell activators/stimulants is needed, being able to precisely supply them into the culturing medium, allowing for different feeding profiles.

### Gas transfer

Gas transfer happens passively in static systems, which limits oxygen availability in high volume vessels, as the diffusive flux of a gas is inversely proportional to the thickness of the liquid that needs to be permeated, according to Fick’s law and the McMurtrey model of oxygen diffusion [60]. Oxygen transfer may be limited in non-perfused bioreactors because low agitation rates are required to minimize shear stress on the lymphocytes and headspace aeration is also generally preferred for the same reasons. This, on the long run, may hinder the final expansion output of the system [41]. .Oxygen can be supplied to a bioreactor either via the headspace or via a sparger which disperses gas into the medium, however, sparging has been shown to be possibly detrimental for immune cell growth [61]. The physiological oxygen concentration is usually lower than the atmospheric. Because of that, establishing culturing protocols that resembles in vivo oxygenation conditions may improve expansion yield and cell functionality [22]. Similarly, the use of CO_2_ levels representative of the biological fluctuation threshold could also be beneficial of the process outcome. It must be noted that reduced oxygen tension results in reduced human T cell proliferation, increased intracellular oxidative damage and susceptibility to apoptosis upon activation, highlighting the importance of controlling oxygen levels in culture [62].

### Physiological congruency

There is no ideal bioreactor that suits all purposes for all cells, but it should be able to replicate in vitro many of the conditions experienced in vivo, therefore it should allow for experimental testing, mechanical conditioning and monitoring of living cells in dynamic conditions [59]. In a close physiological remembrance, immune cells cultured in bioreactors often require APCs for stimulation, three-dimensional culturing, controlled cell-cell contact and undisturbed local microenvironments [25]. These needs should be taken into consideration during the design of suitable devices, starting from the fact that hematopoietic cells do not require a surface to grow, being anchorage independent [63]. It is true that cells can be adapted to a specific bioreactor design as a replacement to engineering the bioreactor itself, but it must be noted that this approach may not be available to most cell therapies, as cells may become senescent after a certain amount of doublings [25]. It should also be noted that some cells may need to be in extensive contact with each other, such as TILs [64] and T cells [65, 66], some of them also tend to form aggregates that must be controlled for optimal growth [67], usually by mechanical disruption of the clusters.

Different reactor configurations may fulfill these requirements to a varying extent. Given this framework, in the next chapter we explore the currently available options and highlight the most relevant characteristics that stand out from comparison.

## Comparison of currently available act bioreactor technologies

During the 1980s, the foundational protocols [68–71] for TILs and LAK therapies were established to be carried out in plates, flasks, bags and roller bottles [72]. At the same time, several attempts of culturing lymphocytes for cytokine production in stirred reactors were being performed [73–77]. It was Knazek [78], Alter [79] and Tanji [80] who in the late 1980s performed the first bioreactor runs intended for cell therapy, using a hollow fiber perfusion system. In the 1990s, the use of the hollow fiber technology increased significantly, while stirred reactors were begun to be used for NK cell ACT applications [81] and the rotating wall bioreactor was introduced as a low shear device [82]. Stirred reactors continued into the 2000s as a solely experimental platform, while the rotating wall technology was not used in clinical applications, focused exclusively in microgravity studies [83–88]. The late 2000s have seen in the usage of the hollow fiber reactor a relative decline compared to the rise in the application of the static culturing G-Rex device (Wilson Wolf Manufacturing, Saint Paul, MN) and the dynamic culturing rocking motion reactor. Both were quickly adopted into clinical practice, stirring the debate of high throughput static vs. dynamic lymphoid cell culturing. In the late 2010s, the hollow fiber reactor returned to wider usage thanks to the Quantum System (Terumo BCT, Tokyo, Japan), and a renewed interest in stirred reactors has been perceived from recent publications [89, 90]. The late 2010s also saw the introduction of the Z RP platform [91] (ZellWerk GmBH, Oberkrämer, Germany) and the Prodigy system [92] (Miltenyi Biotec, Bergish Gladbach, Germany). The latter is an integrated autologous-targeted platform that, despite of its novelty, has been extensively used. There is also high expectation on the Cocoon system [42] (Lonza, Basel, Switzerland) and rotating wheel reactors [11], both announced to be capable of lymphoid cell culturing. Given this historical background, the literature review presented here is based on 117 publications fulfilling the eligibility criteria, of which 73 contained detailed descriptions of the expansion protocols and results, categorized in Rocking motion reactors (16 results), Hollow fiber systems (18 results), Alternative perfusion systems (4 results), stirred reactors (10 results), G-rex-device-based processes (14 results) and Prodigy-system-based processes (11 results). From the 71 articles, 29 contained actual comparisons, mainly between a static protocol and a bioreactor culture with the same stimulation/supplementation strategy.

### Rocking motion bioreactors

In the rocking motion system, a configurable swinging plate conveys a wave-like oscillation to the contents of a culture bag. The continuous agitation ensures proper oxygen transfer and medium homogeneity, which may provide a higher kLa than achievable with a stirred reactor, resulting in greater maximal cell densities under limited oxygen transfer conditions [93]. The agitation pattern is set by the rocking angle and rate, oscillation sequence and culture volume, which translates into a specific fluid flow, mixing time, residence time and oxygen transfer efficiency. This gentle agitation is considered to be a low shear method [94], which may cause lower cell stress even at increased rocking rates, improving nutrient and oxygen transfer efficiency and promoting cell growth without exerting detrimental mechanical conditions to the culture [95, 96]. In contrast to a static system, where cells lay closely together, the continuous oscillation reduces the time that cells may spend in contact with each other, which may not be optimal for cultures requiring close cell-to-cell contact, such as TILs [64] and T cells [65, 66], or adherent cells. Because of that, most cultures performed in this reactor include a static phase prior to the transfer to the rocking platform.

Current rocking motion devices can execute fully automated perfusion cycles, optimizing medium and supplements consumption thus, decreasing the overall process cost. Additionally, perfusion enables cells to be expanded above 10^7^ cells/mL, supporting high volume cultures to be carried out in a single bag with a significantly reduced volume (some bioreactor cultures need about half the amount of media to harvest 10^10^ cells, as compared to static conditions). Since bags are single use there is no need for cleaning validation, they provide a ready-to-use closed system decreasing turnaround time and resource requirements, significantly reducing costs in GMP operations [97]. Consequently, this platform is frequently used academically and industrially during phase 1 and 2 clinical trials [12]. The system also has some disadvantages, including a difficult transition from research scale to full scale GMP expansions. As it is necessary to purchase ancillary equipment additional to the bioreactor [64], it has been argued that rocking motion bioreactors are an ideal solution for scaling the manufacture up from 1 L to 1000 L, but do not economically scale out from one patient to 1000 patients.

The rocking motion bioreactor has been successfully used for T, NK, NKT and TILs expansions (Table 2). Unfortunately, the results of these protocols are not easily comparable due to differences in cell stimulation strategy, media composition, starting material and process parameters. The rocking rate may differ according to the intended application, usually from 5 to 15 rocks per minute (RPM), and a perfusion strategy is generally used, starting from perfusion volume of 250 up to 4000 mL/day. The perfusion begins when a certain threshold is reached either by the decline or increase in metabolites such as glucose, glutamine, ammonia or lactate (especially TIL and T cell cultures) or by the increasing cell density. Once the perfusion is started, the pH and nutrients fluctuate within a narrow range with proven positive effects for TILs and T cells [64, 100], while facilitating glycolysis and glutaminolysis.

**Table 2:** Summary of culture characteristics with rocking motion bioreactors

	Protocol features				Starting material				Reactor configuration				
Author –Year	IL-2 [IU/mL]	Stimulation strategy	Medium	Serum and supplements	Source	Seed concentration [cells/mL]	Bag volume [L]	Culture volume [mL]	System	Rocks per minute	Air flow [L/min]	Rocking Angle [°]	Feeding strategy
T CELLS													
Hami [98] - 2004	Used^a^	CD3 and CD28 beads	Xvivo 15	–	CD3+ T cells from PBMC	–	20	–	Wave Bioreactor System 20XE	–	–	–	Perfusion
Tran [66] - 2007	50	Irradiated PBMCs (APCs)	RPMI	10% FBS; HEPES, GLN and Glucose	CD4+ T cells from PBMC		–	–	Wave bioreactor system 2/10 EH	10–12	0.1	4	Perfusion: Media to keep GLN at 2 mM and glucose > 2 g/L.
Hollyman [99] - 2009	100–500	Pre^b^ - CD3/CD28 beads	Xvivo 15	5% AB	CD3+ from PBMCs	–	2	–	WAVE EHT Bioreactor	6–15	–		Perfusion: Volume increased over 24 h periods (200–1600 mL/day)
Janas [100] - 2015	20 [ng/mL]	Pre - CD3/CD28 beads	Xvivo 10	5% HS; GLN	T cells from PBMCs	5.0 × 10^6^	–	1000	W25 or W5 Xuri cell expansion system	15	–	6	Perfusion: < 2 × 10^6^ cells/mL–0 mL/day; 2–10 × 10^6^ cells/mL–500 mL/day; 10–15 × 10^6^ cells/mL–750 ml/day; > 15 × 10^6^ cells/mL–1000 mL/day
Vavrova [101] - 2016	20	Pre - Ag-mDCs (APCs); CD3/CD28 beads	RPMI	5% AB; GLN, Ne AAs, BME, Pyruvate	T cells from PBMCs	–	2	–	WAVE bioreactor 2/10 system	6	–	6	Media fed: maintain cell concentration in the 0.5–1 × 10^6^ cells/mL range
O’hanlon [102] - 2017	200	Pre PHA	IMDM	10% AB; GLN and glucose	T cells from PBMCs	2.5–5.0 × 10^5^	2	1000	Wave 2/10 (Xuri W5)	5–10	–	–	Perfusion: Culture days 1–2: 250 mL; Culture days 3–5: 500 mL
McCartney [57] - 2019	350	–	Xuri T CEM	5% AB	T cells from PBMCs	–	2	1000	Xuri Cell Expansion System	10	–	6	Perfusion: < 2 × 10^6^ cells/mL–0 mL/day; 2–10 × 10^6^ cells/mL–500 mL/day; 10–15 × 10^6^ cells/mL–750 ml/day; > 15 × 10^6^ cells/mL–1000 mL/day
Smith [103] - 2019	200–500	Pre anti CD3/CD28/CD2	Xuri T CEM	5% HS	Tcells from PBMCS	1.0 × 10^6^	2	–	Xuri Cell Expansion System W25	10	–	6	Perfusion: < 2 × 10^6^ cells/mL–0 mL/day; 2–10 × 10^6^ cells/mL–500 mL/day; 10–15 × 10^6^ cells/mL–750 ml/day; > 15 × 10^6^ cells/mL–1000 mL/day
NK CELLS													
Sutlu [94] - 2010	500	OKT3	SCGM	5% HS	PMBC	2.0 × 10^6^	–	from 800	Wave Bioreactor System 2/10	6	0.1	6	Media fed: 300 mL/day when 3 e6 cells/mL when 7 e6 cells/mL, 500 mL/day; 1 e7 cells/mL, 750 mL/day; 2.5 e7, 1 L/day.
Spanholtz [104] - 2011	Used^a^	GM-CSF. G-CSF, IL-6, IL-7, IL-15	GBGM	10% HS	CD34+ UCB	1.0 × 10^6^	–	from 250	WAVE Bioreactor System 2/10 and BIOSTATH CultiBag RM	10	0.1–0.2	6	Media addition to adjust cell density
Rujkijyanont [105] - 2013	500	CD56- (APCs); IL15 OKT3	SCGM	5% AB	CD56+ from PBMCs	1.0 × 10^6^	2–20	from 200	WAVE Bioreactor	5–9	–	–	Media addition to adjust cell density
Lapteva [106] - 2014	500	K562-41BBL-mbIL-15 (APCs)	SCGM	10% FBS	CD56+ from PBMCs	2.0 × 10^5^	–	–	WAVE Bioreactor	6	–	6	–
Meng [97] - 2018	Used^a^	Pre OK-432	Xvivo 15	1% AP	PBMC	–	3	3000	GE Xuri W25	7	–	6	Perfusion (parameters not specified)
TILs													
Sadeghi [107] - 2011	600	PBMCs (APCs); OKT3	RPMI	5% AB; GLN 12 mM, 25 mM HEPES; BME	TIL	5–10.0 × 10^7^ (TOTAL)	2	1000	Wave Bioreactor System 2/10 (GE)	10	–	6	Perfusion: 350–1000 mL/day to maintain glucose and GLN in a range of 1.5–2 g/L and ~ 2–4 mM, respectively.
Somerville [64] - 2012	3000	Pre – allogenic APCs, anti-CD3	AIMV	5% AB; 0,02% pluronic	TIL/PBL	–	–	1500	WAVE bioreactor 2/10 system	7	–	6	Perfusion: maintain glucose concentration at ~ 170 mg/dL.
Donia [108] - 2014	6000	Pre - allogeneic APCs, antiCD3	AIMV	Pluronic	TIL	–	10		WAVE bioreactor 2/10 system	10	0.2	6	1000–4000 mL/day

^a^Used: stimulant was used but the amount was not specified. ^b^Pre: the stimulant was added to the culture prior to bioreactor expansion

Despite of the difficulties to compare the outcome of different studies, several authors have performed comparative analysis between static set-ups and the conditions provided by a rocking motion reactor (Table 3). In relation to expansion yield, although initially observed as detrimental for growth [94], it has been shown that rocking conditions do not induce significant changes in the total fold of the expansion in case of T cells [66] and NK cells [65, 97, 104], while boosting the growth of TILs [107, 108], DCs and CIK [97]. However, one study found no statistical difference in TIL expansion for static bags compared to a rocking motion bioreactor [64], possibly because of differences in the conditions of media exchange [108]. Similarly, non-perfused T cell cultures has been found to lose viability as low as 80% by the end of cultivation [100] because of critical deprivation of stimulants and metabolites. Contrary to stirred cultures, the use of shear protectant additives has been explored in rocking motion systems, where attempts to expand TIL in the absence of a surfactant (Pluronic F68), derived in significant cell damage and consequent decrease in cell count [64].

**Table 3:** Summary of the results of comparative studies about static and rocking motion cultures

	Expansion yield				Purity		Functionality changes
Author - Year	Static system (vs.)	Static fold^a^	Reactor fold	Culture days	Static	Reactor	
T cells							
Tran [66] - 2007	Bags	247–1340	200–800	14	–	> 98% CD4+	Markers of cell activation increased. No detectable Treg cells produced. Cytokines are produced normally.
NK cells							
Sutlu [94] - 2010	Bags	530	77	20	31% NK	38% NK; 14% NKT	Degranulation and cytotoxic activity are greater in bioreactor cultures.
Spanholtz [104] - 2011	Bags	759–1770	1435–2657	42	71 ± 9% CD56 + CD3-	92% ± 2% CD56 + CD3-	Higher expression of activating receptors in bioreactor cultures 27% degranulation in reactor vs 14–18% in static cultures
Lapteva [65] - 2014	G-rex	No difference		9	Fewer CD3+ T and a higher CD56 + CD3- NK cells in reactor culture		Potency is similar (phenotype and in cytotoxicity assays)
Meng [97] - 2018	Bags	No difference		15	Reactor improves the percentage of NK cells		There is no significant modulation of the cells’ secretome. Cytotoxicity is significantly higher for bioreactor cultures.
TIL							
Sadeghi [107] - 2011	Bags	72 ± 11	228.8 ± 17.1	14	No difference in CD8+ and CD4+ percentage		No difference in Phenotype
Somerville [64] -2012	Bags	1259 ± 137	1130 ± 127	14	Lower CD8 and higher CD4 in reactor		Increased IFN-γ release to cognate peptide in reactor culture Significant phenotype differences
Donia [108] - 2014	Bags	1433 ± 887	5576 ± 1677	14	–	> 97% CD3+	–

^a^Fold = Harvested cells / Seeded cells

Although NK cells’ expansion fold in a bioreactor is the same as in a static system, the proportion of NK cell subpopulations have consistently shown to be enriched under rocking conditions [65, 94, 97, 104]. Reactor-generated products contain fewer CD3+ T cells and higher ratio of CD56 + CD3- NK cells than in static set-ups, perhaps because T cells could prefer non-dynamic conditions [65]. In the same way, clinical-scale activated CD56+ cells in a rocking motion reactor have similar phenotype and function as those derived from static cultures [105]. Unfortunately, the available studies are not clear about the effect of rocking on cell subpopulations in TIL cultures: the phenotype of TIL and genetically modified PBL expanded in static bags and in a rocking motion bioreactor have been found to differ [64]. However, under a different protocol, the numbers of CD4+ and CD8+ populations in a TIL culture were reported to be similar under dynamic and static conditions [107].

In addition to the improvement of the proportion of target cell subpopulation, the functionality of NK cells expanded in rocking motion bioreactors has been found superior than in static systems. Cells cultivated in bioreactors show higher expression of activating receptors such as CD314 (NKG2D) and NCRs, which correlates with a higher degranulation capacity of bioreactor-expanded NK cells (27%) towards K562 cells compared to the 14–18% reached by NK cells in static bag cultures [104]. This higher degranulation profile was also found in a different study [94], as the consequential increase in cytotoxicity [97]. T cell cultures in rocking motion bioreactors have shown increased expression of cell activation markers as compared to pre-cultures [66].

Because of the versatility and successful application of the rocking motion reactor, several studies have been performed using this platform (Table 4). There have been pre-clinical and clinical assays using the rocking motion technology for chronic lymphocytic leukemia [98, 99], metastatic melanoma [109, 110] and prostate cancer [101]. It has also been successfully used to introduce NMR markers during the expansion process [102].

**Table 4:** Further applications of the rocking motion bioreactor

Author - Year	Type	Cell	Disease	Target	Expansion	Functional highlights
Hami [98] - 2004	Pre-clinical	T cell	Chronic lymphocytic leukemia	T cells from Chronic lymphocytic leukemia patients	400 fold in 13 days	High in vitro activity and T cell receptor repertoire restored after expansion.
Hollyman [99] - 2009	Pre-clinical	T cell	Chronic lymphocytic leukemia	T cells from Chronic lymphocytic leukemia patients	87–668 fold in 13–18 days	Transduced and expanded T cells were able to eradicate the tumors in 90% of a mice population; release criteria were met
Andersen [109] - 2016	Clinical	TIL	Metastatic Melanoma	Tumor-Infiltrating Lymphocytes from Patients with Metastatic Melanoma	2856–9975 fold in 13–36 days	Tumor regression was achieved and associated with a higher absolute number of infused tumor-reactive T cells
Vavrova [101] - 2016	Pre-clinical	T cell	Prostate Cancer	Prostate cancer reactive T cell effectors	6 fold in 8 days	Significantly greater cytotoxicity against LNCaP cells after expansion.
Bjoern [110] - 2017	Pre-clinical	TIL	Metastatic Melanoma	Effect of Ipilimumab in metastatic melanoma derived T cells	–	Ipilimumab induced marked changes in T cell infiltrates, which can still be detected despite heavy in vitro expansion.
O’hanlon [102] - 2017	Research	T cell	Non-specific	19F labeling for T cells	–	Cellular viability was maintained; ∼90% of the T cell preparation was labeled with reagent

In regard to on-line monitoring and control, bio-capacitance probes have been successfully integrated into bioreactor bags, and most rocking reactors collect data from single-use DO and pH probes, which can be used, with some limitations, as surrogate measures of VCD to decide on perfusion and DO control, eventually decreasing the frequency of sampling. Alternatively, differential digital holography imaging devices allows for the assessment of cell morphology features and culture characteristics such as cell density, size and viability [11]. Recently, measurements of cellular downstream volatile organic compound (VOC) emissions were made from the gas exhaust lines in a rocking motion reactor, using Headspace Sorptive Extraction (HSSE) and Stirbar Sorptive Extraction (SBSE) coupled with GC–MS. Unique, total VOC profiles correlated well to cell densities over the course of 8 days. The majority of the relevant VOCs decreased during cell expansion that opens the possibility to monitor the nutrients in the media by VOCs and adjust perfusion rates accordingly [57].

### Hollow fiber bioreactors

A perfusion reactor generally uses a semi-permeable membrane to separate cells from the medium. With this technique, culture medium continuously refreshes nutrients and removes waste metabolites in a system that allows specific flow rates on diverse membrane types, making it suitable for continuous cell culture applications, including monoclonal antibody production [111]. This perfusion principle can be achieved with many different membrane systems. However, the most common solution is the capillary-based hollow fiber membrane. In this system, separation occurs as the medium diffuses between the intra-capillary (IC) and extra-capillary (EC) sides and, depending on the maximum size allowed by the membrane’s molecular cut-off, large macromolecules such as cytokines or antibodies are permanently retained on the side where they were originally added [112]. In that way, only small molecules such as carbohydrates, amino acids or small peptides can actually diffuse from and into the compartment where the cells are growing (usually in the EC space), while medium circulates within the IC space [113]. The IC space provides large surface for gas exchange and the cells are not subject to flow therefore they are protected from shear stress [114]. The independent flows in the IC and EC spaces are generated by a set of pumps and valves that direct the fluid through the hollow fiber unit. The basal medium passes through a gas exchange module where sensors are usually placed to monitor parameters such as pH or DO, and sampling systems are allocated for metabolites’ off-line analysis. The flow in the EC circuit generally runs countercurrent to the IC flow, ensuring homogenous distribution of nutrients [115].

Perfusion reduces the need for extensive use of culture vessels and multiple incubators [116]; around 80% decrease in manual labor and incubator space is possible [78]. As the bioreactor uses medium equally or even better than regular static systems [117], up to 30–50 L of medium that otherwise would be used for static culturing [118] may be economically used for perfusion. Furthermore, the cells can grow to high concentrations without the metabolites accumulating in the media. Therefore, cells can achieve the required cell-to-cell proximity for optimal expansion in contact demanding cultures such as TILs. Additionally, multiple therapeutic cell doses can be harvested from a single hollow fiber cartridge, enabling periodic use of the bioreactor [78, 118]. This is also related to the fact that hollow fiber systems are able to support cell growth at densities greater than 10^8^ cells/mL [113]. While a bag cannot handle optimally more than 2 × 10^9^ cells, a hollow fiber reactor could handle at least twenty times that amount [119]. The possibility of executing cell transfection while the expansion is being performed has also been found advantageous by some authors [120–122] as it combines the process of vector concentration and vector-to-target exposure into a single step.

As previously mentioned, the fluid compartmentalization of hollow fiber bioreactors is advantageous due to the decreased physical stress on the cells. However, excessive recirculation of medium may still lead to negative effects [115]. Protein build-up in the EC space does not directly lead to growth inhibition but its accumulation might also limit the convective flow, creating micro-gradients [123]. Meanwhile, reduced microenvironment homogeneity caused by the axial and radial concentration gradients is challenging, as gravity also influences cell distribution within the bioreactor. Some counteract measures, such as periodical rotation, are usually implemented to prevent the cells from sticking together, disrupting the formation of significant detrimental gradients [123]. Harvesting is also not a straightforward process in some configurations, as the detachment and wash out of the cells from the tight pores might require frequent optimization [24] to retrieve as much cells as possible without significantly affecting their integrity and viability; this problem is solved by using a suspension culture configuration, that enables automated sampling and harvesting. In addition, the usually high cell densities attained in the system can be problematic if an electrical or other mechanical issue occurs: the cells do not withstand a decrease in temperature or change in pH as steadily as they do when grown at moderate densities in bags (around 10^7^ cells/mL). It also must be noted that an entire bioreactor has to be harvested to monitor parameters like cytotoxicity, cell phenotype or cell count [72]. A representative sample cannot be periodically obtained from the dense cell culture without performing a major intervention in the system that disrupts the cellular allocation within the fibers while exerts significant shear to remove cells from the thin capillaries, where cell populations are more representative than the cells caught in retention filters. Therefore, culture monitoring is based mainly on physiochemical parameters, or off-line metabolite analysis to the medium effluent, but not on actual cell samples from the culture.

Similar to other expansion systems, it is not easy to compare the performance of different hollow fiber protocols because of differences in the stimulation and culturing strategies (Table 5). The reactor allows the use of cytokines and other growth enhancing additives in high concentration, while significantly reducing the use of serum. Generally, the membranes have a molecular cut-off of 4 to 17 kDa and a very high total surface for optimal diffusion of metabolites. In a typical culture process, cells are seeded at high densities into the EC space, usually after some days of static culture enrichment. But there is at least one exception: the recently introduced Quantum system keeps the cells in the IC space [111, 112], although it allows different seeding configurations depending on the cells to be cultured. The perfusion control strategy is based on the monitoring of the viable cell density by correlating it with non-automated sampling of glucose or lactate concentrations: Glucose consumption and lactate generation rates exhibit logarithmic behavior, correlating with the cells’ doubling time [78, 127]. The culture status may be inferred based on the glucose uptake rates as it reflects the proportion of metabolically active lymphocytes [78]. Although glucose consumption and lactate production rate have been shown to be closely correlated in lymphoid cultures [125], it is possible that lactate levels are not a good indicator of growth inhibiting conditions or nutrient exhaustion, as some cultures seem to grow independently of this metabolite [131].

**Table 5:** Protocol features in Hollow fiber reactors

Author - Year	Pre stimulaiton	Cell culture			IC	Starting material			Culture system					
		Medium	Stimulation	Serum and supplements		Source	Inoculation [cells/reactor]	Volume [mL]	Reactor	Fibers	Cut off [kDa]	Surface area [cm^2^]	Perfusion flow [mL/min]	Control
T cells														
Lamers [124] - 1994	IL2, PHA or CD3 mAb	AIMV	IL2	10% AB	Glutamine and glucose + RPMI	PBMC	10^9^	NP	Immuno*star 4000	10,000	10	NP	50	Control glucose at 1.5 g/L
Lamers [125] - 1999	IL2, PHA	AIMV	IL2	NP	Glutamine and glucose + RPMI	PBMC	1.7–3.1 × 10^8^	100	Immuno*star 4000	10,000	10	NP	50	Control glucose at 2 g/l; control lactate levels
Liu [126] - 1999	Anti CD3/CD8 mAb	CCM	IL2	1% AB	Medium	CD4+ and CD8+ from PBMC	2–8 × 10^7^	NP	Cellco Cellmax	NP	NP	NP	NP	Control glucose at 50–100 mg/dL
Trickett [127] - 2002	IL2, Anti CD3 or PHA	AIMV	CD3/CD28 beads or PHA	5 to 2% FBS	Medium	HIV infected CD4+ cells from PBMC	2–3 10^7^	NP	Cellmax Quad	NP	NP	NP	50	Keep glucose above 50% of the baseline value
De Bartolo [114] - 2007	PHA	DMEM	NP	10% FBS	Medium	PBMC	8 × 10^7^	24	PEEK-WC-HF	NP	NP	128	5 to 10	NP
Curcio [128] - 2012	PHA	DMEM	PHA	10% FBS	Medium	PBMC	8 × 10^7^	25	Parallel-HFMBR	NP	NP	128	2–10	Adjust concentrations to number of cells
Curcio [128] - 2012	PHA	DMEM	PHA	10% FBS	Medium	PBMC	8 × 10^7^	35	Crossed-HFMBR	NP	NP	NP	2–10	Adjust concentrations to number of cells
Nankervis [111] - 2018	IL2, Anti CD3/CD8 beads + IL7	NP	IL2; IL7	NP	Cells	PBMC	NP	100	Quantum 1st generation protocol	11,520	17	NP	EC: 100 IC: 1	NP
Nankervis [111] - 2018	IL2, Anti CD3/CD8 beads	NP	IL2	NP	Cells	PBMC	NP	100	Quantum 2nd generation protocol	11,520	17	NP	Max. 300	NP
Coeshott [112] - 2019	IL2, Anti CD3/CD8 beads	PRIME-XV	NP	NP	Cells	PBMC	3.0–8.5 × 10^7^	124	Quantum	11,520	17	21,000	Max. 300 (IC)	Remove lactate
TIL														
Knazek [78] - 1990	IL2, Autol. LAK supern. and serum	AIMV	IL2 OR LAK supernatant. + HS	Glucose and glutamine	Medium	Melanoma	NP	50	Cellmax 100	8000		11,000	40–300n	Control glucose at 1–1.5 g/L
Hillman [115] - 1994	IL2, TNFa	AIMV	IL2	10% AB	RPMI	Kidney tumors	5–30 × 10^8^	30	IMMUNO*STAR® 1000 Cell Expander	NP	10	3000	EC: 2 IC: 200	Control glucose
Freedman [119] - 1994	IL2	AIMV	IL2	NP	Medium	Ascites/pleural effusions/solid tumors	10^9^	NP	Cellmax 100	8000	4	23,000	60–300	Control glucose
Lewko [129] - 1994	IL2, Autol. LAK supern. and serum	AIMV	IL2	NP	Medium	Tumor	1–2 × 10^9^	NP	Cellmax 100	NP	4	23,000	NP	Control glucose between 1.0–1.5 g/L
Lewko [117] - 2000	IL2, Autol. LAK supern. and serum	AIMV	IL2	NP	Medium	Tumor	1–2 × 10^9^	NP	Cellmax 100	NP	NP	23,000	NP	Control glucose between 1.0–1.5 g/L
Freedman [130] - 2000	IL2	AIMV	IL2	NP	Medium	Tumor samples	1–2 × 10^9^	NP	Cellmax 100	NP	NP	NP	NP	NP
Malone [118] - 2001	IL2, OKT3	AIMV	IL2	Glutamine	Medium	Tumor samples	4–6 × 10^8^	100	Celco - not specified	NP	30–150	2200	NP	Keep lactate below 1000 units/mL
PBL														
Pan [120] - 1999	IL2, OKT3	AIMV	IL2	5% FBS, glutamine	Medium	PBL	5–9.3 × 10^7^	NP	Cellmax Quad pump station	NP	NP	NP	NP	Controlling lactate levels
Shankar [121] - 1997	OKT3	AIMV	IL2	5% FBS, glutamine	Medium	PBL	5 × 10^7^	11.4	Cellmax Artificial Capillary	NP	NP	NP	NP	Keep lactate below 0.5 mg/mL
Stroncek [122] - 1999	OKT3	AIMV	IL2	5% FBS, glutamine	Medium	PBL	10^8^	11	Cellmax Artificial Capillary	NP	NP	NP	NP	NP

*NP* Not published

Initial studies have not found a significant difference in the expansion yield of cells cultured in the hollow fiber bioreactor as compared to the classical static culturing methods [78]. However, later experiments shown conflicting results in this regard (Table 6). Cell expansion is usually in the range of 100 to 200 fold after 1 to 2 weeks of culturing, but newer technologies have reached higher than 500 fold after 8 days [111, 112]. These results should be weighed against the seeding characteristics and the specific stimulation strategy. It also seems that culture performance cannot be easily predicted based on viability or inoculum density [129] as total medium consumption differs between cell donors as a function of the metabolic activity of their cells [78]. Even when the culture performance is highly variable, a lag phase (lasting from 1 to 9 days) is generally observed [117, 119, 129], then the glucose consumption and lactate production rate change exponentially reaching a plateau or peak after some days of culturing [121, 124]. More pronounced peaks for lactate generation can be seen for high seed cultures, followed by a faster decrease than observed in low seed cultures [112]. The lactate production may start to increase right after inoculation when a static pre-adaptation is performed [120]. Different patterns of cell-produced cytokine concentrations can also be observed during T cell expansions, as TNFα, IL-6, IFNγ and GM-GSF are proportional to the extent of lymphocyte multiplication, which may depend on cell donor and to the formation of microenvironments, hindering the supply of nutrients and oxygen to some cultures [125].

**Table 6:** Performance differences between static and perfusion reactor cultures

Author - Year	Expansion					Purity	Functionality changes
	Fold	Days	Static control	Fold	Days		
T cell							
Lamers [124] - 1994	52.6 ± 21.3	14–17	Bag	238.4 ± 168.7	14–17	CD4/CD8 ratio = 0.51 ± 0.23 vs. CD4/CD8 = 0.44 ± 0.16 static culture	NP
Trickett [127] - 2002	53.2 ± 20.1	7–8	Flasks	71.2 ± 42.8	7–8	NP	NP
Jones [60] 2020	17.7 fold higher than static	9	Flask	–	9	Treg phenotype 93.7% for flasks versus 97.7% for reactor.	Reactor cultures had 8-fold greater interleukin-10 stimulation index
TIL							
Knazek [78] - 1990	124–1170	14–32	Bag	No difference	NP	NP	Bag and hollow fiber cultures has similar surface-antigen profiles; Cytotoxicity was similar in both systems
Hillman [115] - 1994	20–60	7	Plate	3	7	Shift in the T cell subpopulations is more pronounced in the bioreactor.	NP
Freedman [119] - 1994	30.6 ± 5.6	18.2 ± 1.7	Plate, flask, bag	303.1	28.9	CD4/CD8 ratios do not have a statistically significant difference; no difference in proportions of CD16+ and CD56	NP

With respect to product purity, cultures grown in hollow fiber bioreactors have shown to consistently achieve high levels of target cell fraction (Table 7). T cells grown from TILs and PBMCs do not have statistically significant differences in their CD4+/CD8+ ratios [119, 124], however it has been reported that CD8+ T cells prefer to expand in low-seed cultures, while CD4+ T cells expand more in high-seed cultures [112]. Furthermore, certain shifts in T cell subpopulations can be higher in bioreactors compared to static cultures [115]. The proportions of CD3+ cells may also increase throughout TIL expansion processes [119]. In general, the stimulation strategy has a greater impact on the cell differentiation profile than the culturing platform, because the stimulation protocol is specifically designed to induce a specific phenotype and may only be enhanced by the direct contact of the cells with the stimulant, which corresponds to the nature of the system. In the same way, bag and hollow fiber cultures have shown similar surface antigen profiles and cytotoxicity [78] with normal cytokine production profile [129] and no functional alteration upon re-stimulation as measured by IFN-γ, IL-2 and TNF-α secretion [112]. Reactor grown cells also preserve the same biological properties as those grown in static set ups [115] and T-cell products had lower abundance of exhaustion markers [112] when grown in the Quantum System. However, a reduction in cytolytic activity at the end of the culture has also been described [125] and an increase in the concentration of cytokines or growth factors in the medium, produced by the PBLs has been proposed as a reason for overshadowing any inhibitory effects related to the increased lactate levels [122]. There are also alternatives available for perfusion reactors that were not discussed here in details but were used previously for ACT manufacturing (Table 8).

**Table 7:** Performance of non-comparative cultures in hollow fiber reactors

Author - Year	Expansion			Purity	Functionality changes
	Fold	Days	Media feed [L]		
T cell					
Lamers [125] - 1999	41–149	15	NR	Predominantly CD3+ and CD8+	Reduction in cytolytic activity at the end of the culture
Liu [126] - 1999	2 × 10^5^–10^8^	50–70	NR	95–99% CD4 + CD3+ T cells with virtual elimination of CD8+ cells	50–95% of the cells had elevated expression of HLA-DR.; (IL2R)-a chain expression was increased; 40 to 90% of CD25 levels higher than freshly isolated CD4+ T cells
Nankervis [111] - 2018	117,450 (1st generation)	13	2.2–2.4	90.9–98.8% CD3+	NR
	439–557 (2nd generation)	10	10.4–13.9	98.8–99.5% CD3+	NR
Coeshott [112] - 2019	543–1079 (high seeding)	8	19.9	91.9–94.5% CD3+; CD4+ expanded preferentially	T-cell products had had low frequencies of cells bearing exhaustion markers
	951–1787 (low seeding)	9	13.6	94.2–97.5% CD3+; CD8+ expanded preferentially	
TIL					
Lewko [117, 129] - 1994	17.3	22.3	40	96% T cells based on CD2+ reactivity.	Cells produced cytokines normally
PBL					
Pan [120] - 1999	104–187	11–12	NR	NR	57% transduction frequency
Shankar [121] - 1997	~ 100	10	5.5	NR	1–10% transduction frequency
Stroncek [122] - 1999	~ 200	17	NR	NR	< 2.5% transduction frequency

**Table 8:** Alternative perfusion reactors for ACT

		Culture			Results					Characteristics		
System	Cells	Medium	Stimulation strategy	Perfused medium	Inoculated cells/ml	Days	Yield	Purity	Functionality	Features	Disadvantages	Other ACT
Aastrom	TIL [132]	AIMV, RPMI 1640, HEPES, BME, 10% AB serum, 6000 IU/mL IL-2	Irradiated PBMC APCs; 6000 IU/mL IL2, OKT3	AIM V, 1% human serum, Glutamine 6000 IU/mL rhIL-2	5 × 10^6^	14	up to 5.8 × 10^9^ cells (1127 fold)	Populations nearly identical to static; 90% CD8+	Activity against HLA-A2+ matched tumor lines; IFNγ secretion equal or higher than in static cultures	Slow medium exchange rates maintain a tissue-like microenvironment	Scaling is not available; limited opportunity for in-process monitoring; low surface area generates low yield	UCB [133]; DC [134]
ZRP	NK [91]	Alpha medium, glucose, 10% HS, glutamine, 1000 IU/mL IL2; proprietary activation cocktail.	Specific proprietary activating cocktail	NP	70 × 10^6^	12–22	~ 14 fold	NK cell purity > 85%; T cells, B cells and NK T cells were below 2%	Cytotoxicity did not exceed 20% expression of activating receptors; strong IFNγ expression	Directed laminar flow of medium, which allows an undisturbed cell/cell- and cell/surface-contact and minimizes cell stress	Efficiency and killing capacity are questioned	Other NK [19]
	NK /γδT /CIK [135]	RPMI1640 complete medium (10% HS and 100 IU/mL IL2)	Irradiated K562- mb15–41BBL cells	NP	10^6^	14	Static culturing resulted in higher cell counts than Z®-RP	Majority of expanded NK/γδT/CIK cells developed a CD56 bright phenotype	Static culturing resulted in higher cytotoxicity of NK/γδT/CIK than in dynamic culturing			
Packed Bed	Tonsil tissue cells [136]	OPTI-MEM; 7.5% human AB serum	NP	OPTI-MEM	10^7^	NP	Tissue formation	NP	NP	Architectural features typical of lymphoid organs	NP	DC [137]

### Stirred bioreactors

While hollow fiber reactors focus on highly efficient and compact cultures, rocking motion systems specialize in easily scalable platforms, the stirred reactor, as the most widespread and classical bioreactor technology, excels in tight process control and straightforward scale up due to easy parametrization, ideal for process intensification. These bioreactors are characterized by a central agitation element, which keeps the medium in motion, thereby maintains cells and stimulants in suspension and provides homogeneous distribution of gases and nutrients [35]. The vessel’s geometry, the shape of the impeller and the selected mixing and aeration strategy influence the culture’s yield and cell surface markers expression [138]. This translates into a versatile system with high process control capability [89], that provides an efficient mass transfer of oxygen and nutrients, high robustness, precise process control and outstanding scalability. These features enabled stirred reactors to be the first platform employed for lymphoid cells culturing. It was initially used for lymphokine production [73–77], although it was later replaced as more efficient techniques were available for cytokine manufacturing. After that, diffusion of stirred bioreactor into cell therapy was slow and mainly circumscribed to small-scale experimental applications. The spinner flask has been frequently used in that regard, as the simplest stirred vessel, having a couple of side-arm vents for gas and medium exchange and a central stirrer shaft [24]. This reactor is often used as the first step to adapt new cell types to stirring [89]. Culturing cells for ACT in stirred reactors is mainly useful in allogenic therapies, where process scale-up is more important, contrary to scale out primacy with patient-specific applications [11].

As hematopoietic cells are relatively sensitive to shear, the mechanical stress induced by impellers has become a main concern when using a stirred reactor. In that regard, higher than 75 rpm has been found detrimental for some T cell cultures [139], but such low shear rates may unlikely to produce physical damage to the cells and it is more plausible that the cells actually respond to the transduction of fluid-mechanics forces at a molecular level [63]. Additionally, a decrease in the rate of proliferation has also been observed when gas sparging is used instead of surface aeration, as rupturing bubbles may subject the cells to hydrodynamic forces that could affect the expression of the IL-2R receptor [61]. This receptor has been frequently found to be downregulated in cultures subjected to stirring conditions [139, 140]. In addition to the proved downregulation of IL-2R, agitation could include effects such as changes in gene and protein expression, disturbances in plasma membrane permeability and cell cycle and changes in other intracellular signal pathways [141]. Due to the enhanced interaction between the cells and the stimulant agent, demonstrated increase in cell expansion and phenotype at high stirring levels with cultures that used stimulation beads for cell activation [89]. Although it has been suggested before [61], the use of shear protectant additives has not been investigated yet in stirred reactors. These additives may also prove useful in countering the negative effects observed on the IL-2R downregulation.

Stirred reactors have been applied for expanding T and NK cells, although cell-to-cell contact-intensive cultures have not been successfully executed yet. Protocols are different (Table 9) but there are several common points. As previously mentioned, almost every protocol use a low stirring range between 50 to 70 rpm. The seeding density is usually below 1 × 10^6^ cells/mL and the culture is kept at a low cell density throughout the duration of the expansion, implying a very high final culture volume to attain clinically relevant cell counts on the long run. Cell retention by filters has also been applied for stirred vessels [144] but with no remarkable differences from non-perfused cultures. DO levels are set into a 5 to 70% wide range. Interestingly, hypoxic conditions have frequently been found ideal for cell growth [140, 145, 146] as the best cell expansion is usually obtained by culturing at the lowest oxygen tension. This phenomenon could be explained by the low mean O_2_ tension in the hematopoietic and lymphoid organ tissues, that is closer to 40 mmHg (or 5% O_2_ in the gas atmosphere), while the anatomical architecture of these organs might expose cells to even lower O_2_ tensions [145]. In a similar manner, maximum T cell growth rate has been found to increase at 38.5 °C, although most of the published culturing protocols used 37 °C [140].

**Table 9:** Protocol features for stirred systems

Author	Protocol			Stirred system						Expansion		Static control		Purity	Functionality
	Stimulation strategy	Medium	Key Supplements	Vessel	Stir. speed [rpm]	Aerat. rate	DO	Seed density [cells/mL]	Volume [ml]	Fold	Days	Fold	Days		
T cells															
Ou −2019 [90]	IL-2;anti CD3/CD28 beads or mABs	OpTmizer CTS	Serum-free	2 L Stirred vessel	70	0.01 vvm	70%	5 × 10^5^	800	132–1011	4	–	–	> 99% CD4+ and CD8+ T cells	High CD3 expression (91.7–99.4%); T cell co-stimulatory signaling receptor (ICOS/CD278) elevated; upregulated expression of PD-1/CD279; production of IFNγ decreased by ~ 50%
Costariol – 2019 [89]	IL-2; anti CD3/CD28 beads	RPMI 1640	10% FBS; Glutamine	Spinner	35	–	–	5 × 10^5^	100	No growth	–	–	–	CD4:CD8 ratio decreased from 4:1 to 1:1	–
				ambr 250	100–200	14.25 mL/min 21% O_2_	60%	5 × 10^5^	250	23.2 ± 1.3	7	15.2 ± 3.1	7		Effector memory cells increased from 35.69 ± 10.98% to ~ 80%; no significant difference in T cell subpopulation profiles between static and ambr® 250
Ramsborg – 2004 [142]	IL-2; anti CD3/CD28 mABs	AIM V	2% AP	Spinner	60	–	–	1.5 × 10^5^–3.0 × 10^5^	100	5.4 (0.5–87)	15			CD3 cells > 90%; CD8 cells preferentially expanded over CD4 cells	–
Foster – 2004 [143]	PHA or OKT3; 50 IL2;	RPMI 1640	10% AB; Glutamine, HEPES, pyruvate	Spinner	50	–	–	5 × 10^5^	100	> 10	7	> 10	7	NLV–tetramer + CD8+ phenotype > 95%	CD25 decreased exponentially. This behavior did not vary significantly between suspension and static cultures;CTL maintained their specificity toward CMVpp65
Bohnenkamp – 2002 [140]	IL-2; pre anti CD3/CD28 mABs	aMEM	10% FBS	Spinner	–	–	–	–	–	394	9	–	–	> 94% CD3+ cells; CD4:CD8 from 2.4:1 to 1:5 (stirred vessel), 1:2.7 (suspension bioreactor) and 1:4.8 (T-flask)	–
				1 L stirred vessel	–	–	–	1.35 × 10^5^	–	44	10	Not different	–		IL-2R was downregulated earlier than in the static T-flask culture; IFNγ secretion assay against a hCMV protein maintained
				Stirred 50–550 mL	–	–	–	5 × 10^5^	–	60	10	Not different	–		–
Hilbert −2001 [144]	IL-2;anti CD3/CD28 mABs	RPMI 1640 OR AIMV	10% FBS; Glutamine	18 L stirred vessel	–	–	–	–	18,000	11	10	No difference		> 90% T-cells	–
				550 mL cell retention reactor	–		20–50%	3.8 × 10^5^	550	67	5	–	–	–	–
Carswell – 2000 [145]	IL-2, pre PHA or antiCD3	RPMI 1640	10% FBS; Glutamine; pyruvate, neAAs	Spinner	60	–	5%	–	100	1214 ± 374	16	–	–	–	–
NK cells															
Pierson – 1996 [81]	IL-2; pre anti-CD5/CD8 mABs	(DMEM)/Ham’s F12	10% FBS; ascorbic acid; selenite	Reactor	60	–	40%	10^6^	530	352	33	–	–	> 90%	NK-specific cytolytic function maintained
				Spinner	60	–	–	10^6^	250	10^7^ ± 17	28	43 ± 11	28	86 ± 9,5% CD56+/CD3-; similar to static	75% specific lysis of K562; similar to static

Productivity-wise, and probably because of the operational limitations to avoid any possible damage inflicted by impellers, T cells cultured in stirred reactors experience little [89, 140] to insignificant [61, 139, 144] boosting in their proliferation as compared to static systems. T cell differentiation have also been found not to be impacted by the agitation regime, with a similar phenotype to static controls [89]. As with other expansion technologies, there is a high expansion variability for cultures processed under the same conditions, likely due to raw material variability. Contrary to T cells, stirred bioreactors have been found to increase the total NK cell production by 7 fold compared to static cultures [81], however the application of this kind of reactor to NK cells has not been further explored and there is need on additional comparable results to conclude on its potential. Similarly, Peripheral Blood Mononuclear Cells (PBMCs) cultured with 30 rpm stirring speed have shown comparable [147] or superior [148] expansion levels than in static systems. As a comparison with similar cells, cord blood derived hematopoietic stem cells (CB-HSC) have also been found to better expand in stirred systems than in static culture, when agitated between 30 to 40 rpm [149, 150]. They also present a different expression of genes mainly responsible for chemotactic activity DNA repair and apoptosis [151]. Stirred reactors were also tested for ex-vivo expansion of encapsulated primary human T lymphocytes, but growth rates were lower in dynamic conditions [152].

The possibility to develop robust control strategies in the ACT field would be one of the main advantages of stirred bioreactors; however, little research has been published in this regard. Pierson et al. [81] tested on-line laser turbidity measurement that reportedly correlated well with cell counts. Recently [46], T-cells cultured in stirred vessels fitted with Raman probes were used to develop chemometric models for glucose (*R* = 0.987), lactate (*R* = 0.986), ammonia (*R* = 0.936), glutamine (*R* = 0.922), and glutamate (*R* = 0.829). Univariate Raman modeling for non-targeted analysis of the culture media was found useful to track the nutrient depletion (glucose and glutamine) and metabolite production (glutamate and lactate), with similar accuracy to the chemometric models. Despite of that, no further research on the application of advanced process analytical technology for stirred cultures in ACT has been done. Manual sampling, coupled with at-line and off-line measurements is routinely performed to measure other process parameters such as cell density, viability, and metabolites concentrations [11]. The lack of process understanding has prevented the development of suitable mechanistic models which still have major discrepancies between predictions and experimental data [153].

### Culturing platforms specialized on ACT

Besides traditional bioreactors, derived from long established bioprocess applications, some expansion technologies were developed to specifically address the requirements of autologous and allogenic ACT. These platforms aim to either provide a physiological-like environment, or to efficiently integrate, from cell acquisition to product formulation, the complex cell therapy workflow into a robust and GMP compliant automated system. Although these platforms have become available just in the last decade, they have been extensively and successfully tested. They are already implemented in clinical practice and cell culture processes, that will be discussed below, are evolved around the devices themselves, hence the different processes are categorized by the culturing platforms they were performed on.

#### Processes with the G-rex flask

The G-rex flask is a cylindrical vessel, equipped with a silicon membrane for gas exchange, that enables the usage of a great amount of medium without requiring mechanical assistance for oxygen transfer [106]. Its geometry allows for a set of linearly scalable vessels with a surface area from 5 to 500 cm^2^ [154], starting from permeable six well plates [155, 156], up to 4500 mL flasks. The increased medium quantity, usually limited by superficial gas diffusion to the cells, supplies nutrients and allows waste dilution into a greater volume, while enhancing close cell-to-cell contact [157], however, the final cell density in a G-rex flask is mainly limited by gas exchange rather than by exhaustion of nutrients [158]. The device includes an automated harvesting unit that allows to perform the expansion in a fully closed system [10]. The G-rex favors differential expansion of specific cell subsets, as it allows oxygen-demanding cells to better survive and proliferate with a more oxidative phenotype and higher levels of mitochondrial activity [159]. Furthermore, it could help to rescue certain lymphocyte lines that can poorly grow in traditional culture devices [159, 160]. Because of the static culture environment, G-rex bioreactors excel in protocols that use APCs such as TILs and antigen-specific T cells [11].

Compared to static systems, the G-rex decreases the amount of manual labor by approximately four times [158] and shortens manufacturing time of some T cell protocols by half [161]. Even in protocols that couple transfection and expansion, the materials’ cost is approximately 38% less than in a bag-based process [162]. As the G-rex can be used for pre-REP operations, traditional flasks can be entirely eliminated from the manufacturing process [106]. On the other hand, cell expansion kinetics is affected if the cells are disturbed, hindering process sampling [12]. The G-rex flasks cannot incorporate real-time visualization of the cell culture because of their standing nature [13]. Unfortunately, even when the G-rex process is scalable, there is still a need for using several flasks to get an adequate number of cells for treatment, increasing cost and workload [33]. However, the versatility of this device has allowed its application to diverse ACT fields: the manufacturing of multi-virus-specific [163–166], adenovirus-specific [167], cytomegalovirus-specific [168] and EBV-specific [169] cytotoxic T-cells for infection control after hematopoietic cell transplantation. Furthermore, the GMP manufacturing of NKG2D CAR-T cells for acute myeloid leukemia and multiple myeloma [170], gene-edited NK-92 and YTS cell lines expansion [171] and the generation of TIL for ovarian epithelial cancer [172] were also made possible.

Most of the applications of the G-rex extensively use feeder cells (Table 10), profiting on the enhanced close cell-to-cell interaction. The platform allows expansion levels above 2000 fold for TIL, T cell and NK cell within 2–3 culturing weeks using highly standardized protocols for GMP-grade cell manufacturing. That includes peer-reviewed guides for CTL from PBMC [182, 183] and UCB [184], NK cells [185], TILs [186] and CAR-T cells [157]. When comparing its yield, the expansion of T cells in the G-rex generates up to 100 [173] to 1000 [158] times more cells than classical static culturing techniques, but there are lower yield exceptions [175, 177]. In the same way, NK cells and TILs have also shown better or similar expansion performance, but the difference is moderate [106, 159, 180]. Processes in the G-rex flask also achieve good results regarding cell purity and functionality. The CD4/CD8 ratio of T cells cultured in the G-rex flask tend to be preserved [158, 160, 162], while the target cell purity is generally above 90% [158, 173, 174]. NK cells have also been produced with above 95% purity, even in complex protocols derived from Umbilical Cord Blood [179]. In TILs, the Phenotype has been found to be similar to static systems [180], with no evidence for cloning selection [159]. Functionality-wise, T cells grown in the G-rex preserve their cytolytic activity [158, 160, 176] and some research have found better [177] to similar [162] cytokine expression profile compared to static controls. The cytokine production of TIL grown in G-rex flasks is similar to TIL produced with 24-well plates, T-175 flasks, and bags [180].

**Table 10:** Protocol features for G-rex systems

	Protocol features				Starting material			Expansion			Comparator			Purity	Functionality changes
Author (ref) - year	IL-2 [IU/mL]	Stimulation	Medium	Suppl.	Source	Seed [cells /mL]	Vol. [mL]	Culture system	Fold	Days	System	Fold	days	Target	Cytotoxicity
T cell															
Vera [158] - 2010	50	Irr. EBV-LCLs	RPMI; CM	10% FBS; glutamine	T cells from PBMC	5 × 10^5^	30	G-rex40	1700–2200	23	plates	3–5	23	No change in phenotype; > 90% CD3+ cells (96.7 ± 1.7 vs 92.8 ± 5.6; G-rex vs 24-well), CD8+ (62.2% ± 38.3 vs 75% ± 21.7).	Cytolytic activity maintained, killing vs EBV-LCL 62% ± 12 vs 57% ± 8; G-rex vs 24-well plate
Chakraborty [173] - 2013	50	Anti CD3/CD28, temsirolimus, Irr. APCs	RPMI	10% AB, glutamine BME	nTregs from PMBC	NP	400	G-rex100	> 600	21	plates	5–6	21	Percentage of CD4 + CD25+ remained stable at 92 ± 5% by day 21	FoxP3+ cells increased from day 1 to day 21 of culture. No compromise in telomere length.
Gerdemann [174] - 2013	NP	Irr. nucleofected DCs, IL4 IL7	RPMI; CM	10% FBS; glutamine	rCTLs from PBMC	2 × 10^7^	30	G-rex10	7–28	9–11	NP	NP	NP	CD3+ T cells (mean 98.6 ± 0.1%), CD8+ (59.6 ± 2.7%) CD4+ (34.1 ± 2.5%)	CTL lines specific for EBV, CMV, and Adv antigens but not alloreactive.
Ramanayake [175] - 2015	50	Irr. Autologous PBMCs or Nalm-6 cells; IL15	AIMV	10% AB OR FBS	T cells from PBMC	10^7^	NP	G-rex10	680.4–765.4 fold	22	plates	326.8–576.0 fold	22	57% CAR expression (range 50–63%) in G-rex10 vs. 66% (35–78%) in plates; CD3+ in G-rex10 88% vs. 96% in plates	NP
Orio [160] - 2015	NP	IL-4, IL-7, IL-21	RPMI; CM	10% AP, glutamine	T cells from PBMC	2 × 10^7^	NP	G-rex10	9.2	12–14	NP	NP	NP	Balanced CD4/CD8 T-cell ratio, slight CD8 predominance.	Antigen-specific, allo-tolerant, anti-EBV T-cell lines
Jin [176] - 2018	3000	Irr. allogeneic PBMCs; anti-CD3	AIMV	5% AB, glutamine	T cells from PBMC	10^7^	800–4500	G-rex500 MCS	1890 ± 138	21	NP	NP	NP	CD4+ T-cells were favored transduction efficiency of 74 ± 10% and 93 ± 1.4%	Transduced T-cells lyse CASKi cells (48 ± 4%) and 293 cells expressing E6 (72 ± 1%). E6 and E7 TCR transduced T-cells produced IFN-γ and TNF-α.
Kuranda [177] - 2019	Used	GM-CSF, IL-4, IL-1b, Flt3L, TLR8L, TNF-a, PGE2, IL-7, IL-2, IL-15, IL-7.	AIMV	10% HS	Ag or AdV5-reactive CD8+ from PBMC or CB	10^7^	NP	G-rex10	11 fold vs. std.	10	NP	NP	NP	NP	More CD8+ T cells producing TNF-α, IL-2, IFN-γ, and/or MIP-1b for G-rex cultures. IFN-γ, and MIP-1b were undetectable in plates.
Gagliardi [162] - 2019		Viral supern. and Vectofusin-1; IL7, IL15, antiCD3/CD28	TexMACS GMP	10% FBS	Transd. T cells from PBMC	NP	1000	G-rex100 MCS	Higher than bags		Bags			No difference in CD45RA and CD62 L cell expression or CD4:CD8 ratio	Transduction in G-rex 55 ± 7%, vs. 73 ± 7% in bag. More viable and transgenic cells from G rex. Secretion of cytokines did not differ.
Xiao [178]- 2018	300	Zometa; Irr K562 Clone A APCs; OKT3	AIMV	5% AB	Vγ9Vδ2T cells from PBMC	NP	NP	G-rex10 & 100	10,995 ± 3078	17	NP	NP	NP	The Vγ9Vδ2T-cell purity achieved was 85.98% ± 10.28%. The NK population decreased from 20 to 4.6%	G-rex promoted phenotypic changes: TEFF cell decreased from 66.6–51%; TEM cell increased from 21 to 37.6%. around 40–80% target cell killing.
NK cell															
Lapteva [106] - 2012	10	Irr. K562-mbIL15-41BBL cells	SCGM	10% FBS; glucose	CD56+ CD3-NK cells from PBMC	2 × 10^6^	400	G-rex100	442 ± 29	10	Bags	227 ± 91	10	Less than 35% proliferation of T cells was detected during the NK cell expansion.	NP
Shah [179] - 2013	100	Irr. APC cells	RPMI; CM	10% AB	NK from Cryopreserved CB	NP	NP	GP500	2389	14	NP	NP	NP	95% purity for NK cells (CD56+/CD32) and less than 1% CD3+ cells.	Significant in vivo activity against MM in a xenogeneic mouse model
TIL															
Jin [180] - 2012	3000	Irr. allogeneic PBMC, anti-CD3	AIMV	5% AB	Tumor fragments or tumor digest cells	5 × 10^6^	400	G-rex100	176 + 136	7	T175	167 ± 37	7	Phenotype similar to TIL produced with 24-well plates, T-175 flasks, and bags.	IFN-γ similar to T-175 flasks and bags (Table 4).
Forget [181] - 2014	Used	Anti CD3; Irr. allogeneic PBMC OR Aapc	RPMI	10% AB, glutaminepyruvate, HEPES, BME	Melanoma tumors	5 × 10^6^	NP	G-rex100 M	> 2000	NP	NP	NP	NP	Mostly CD3+ cells for both conditions	Decreased CD28 expression in T-flask system was not observed when using the G-rex flasks
Forget [159] - 2016	Used	anti-CD3, Irr. allogeneic PBMC		NP	Melanoma tumors	5 × 10^6^	400	G-rex100 M	2653	14	Plate + bag	1210	14	G-rex did not favor clonal selection	No Va or Vb expression was lost with any system. TCR diversity was not altered. The OCR of the TIL in G-rex almost tripled, demonstrating an enhanced mitochondrial capacity

#### Processes with the prodigy system

The Prodigy® system integrates cell washing, separation, enrichment and expansion into a fully automated GMP compliant workflow. Prodigy’s culturing function is executed by a centrifugation chamber equipped with a microscope camera [92]. The system is able to control temperature, DO, pCO_2_ and it can exchange media while performing detailed stimulation protocols using a single set of tubing [92, 187]. The expansion chamber switches from static to dynamic culturing, applying short centrifugation pulses in order to gently mix the cells after stationary intervals that promote cell contact and clustering [188]. Its integrated enrichment and separation functionality has been successfully tested for CD34+ hematopoietic stem cells [131, 189, 190], T cells [191–194] and NK cells [195, 196], decreasing the risk of contamination and increasing process consistency, while reducing personnel and processing time [187], behaving essentially as a “walk away” process for most of the culture period. However, the Prodigy system was designed to be fully closed and automated, rather than to maximize cell expansion. This characteristic is a limit to use the system for protocols requiring large amounts of cells [197]. Thus, if a vast amount of lymphocytes is required for infusion, there will be a need to perform several expansions in multiple devices for a successful therapy [198]. It is expected that new tubing sets with enhanced culturing volume, or the integration of a higher scale bioreactor within the platform [198] could enable a fully automated system that is able to scale from an initial expansion phase to a late cultivation stage at higher culture volumes.

Most of the protocols developed for the Prodigy system couple gene transfer and expansion (Table 11), reaching transduction efficiencies in the range of 20–30% [200, 201, 203], 50–60% [197, 202, 204] and 80% [199]. Compared to rocking motion cultures, that require a pre-cultivation phase to generate enough cells, a lower cell amount is needed to inoculate an NK [198] or T cell culture [200, 201, 205]. The Prodigy-based lymphoid cultures have shown similar growth kinetics to static systems, such as the G-rex [205], but final yield is usually lower [197, 198]. The maximum increase in cell number is generally below 50 fold after 10–13 days of culturing [187, 188, 197, 199, 202, 204] but there are some higher yield exceptions [200, 203]. Despite of this, over short periods of time, the observed fold expansion is significantly higher than traditional methods, indicating a stable advantage of high yield in brief expansions [202]. NK culturing protocols have consistently reached target cell fractions around 99% [188, 198] and T cells have been produced at around 95% purity [187, 199, 203, 205]. The phenotype profiles of automatically and manually expanded cells have been found to be similar in NK [198] and T cells [197]. Gene expression analysis has shown just slight divergences between NK cells expanded manually or through automation and a similar IFN-γ expression in automated and manual NK cultures have been reported [198]. However, IFN-γ has also been found to be decreased in Prodigy-based T cell cultures compared to static protocols [187, 197]. Despite this, the cytotoxicity of products from a Prodigy system have been generally found to be compliant for their clinical application [187, 188, 197, 201–204].

**Table 11:** Protocol features for Prodigy systems

Authors	Protocol features				Starting material			Culturing		Expansion				Purity	Functionality
	IL-2 [IU/mL]	Stimmul. strategy	Medium	Serum	Source	Seed [cell/ml]	Vol. [mL]	Tubing Set	Shaking	Fold	Days	Compared system	Comp. Fold		
T cells															
Mock [187] - 2016	Used	CD3/CD28; Transd.	TexMACS GMP	3% HS	T cells from PBMC	7.45–10 × 10^8^	100	TS520	Shaker from day 4/5. More vigorous shaking depending on cell density	16.2 ± 7.9	8–10	G-rex 10	22.3 ± 12.2	No difference with G-rex;98.0% max T cell purity	Exhaustion profile did not differ. Prodigy cells produced less IFN-γ. Secretion of TNF-α and IL-2 was lower but not significant specific lysis to target
Priesner [199] - 2016	Not used	CD3/CD28; Transd.; IL7; IL15	TexMACS GMP	3% HS	CD62L+ cells from PBMC	3 × 10^9^ total	NP	TS520	Static culture until day 3; clusters were dispersed	13–23 (cells); 28–42 (Tcells)	10–13	Not used		83.4–98.9% CD3 + CD45+ T cells	Transduction efficiency of 83%
Lock [200] - 2017	Not used	CD3/CD28(transact); Transd.; IL7; IL15	TexMACS GMP	3% HS	CD4 + CD8 + CD45+ from PBMC	2–10 × 10^7^ total	250	TS520	Automated media exchange every day	65 ± 36	12	Not used		91.3–5.0% for Healthy donor and 88.3–7.1% for Melanoma Patient sourced	Transduct.: 34.5–11.7% for HD and 36.4–17.7% for PM. Secretion of GM-CSF, IFN-γ, IL-2, and TNF-α, no IL-4, IL-5, or IL-10 detected.
Blaeschke [201] - 2018	Not used	CD3/CD28(transact); Transd.; IL7; IL15	TexMACS GMP	3% HS	CD4+/CD8+ from PBMC	2.86 × 10^7^ total	NP	TS520	NP	14.7–102.4	12	Not used		50.3% CD4+ and 38.7% CD8+ cells; 10.2% of the cells were NKT cells.	Transduction: 26.95%; No increased expression of exhaustion markers. CD19 CAR T cells killed 80% of the target (5:1); cells were able to secrete GM-CSF, IFN-γ, IL-2, and TNF-α
Zhang [202] - 2018	200	CD3/CD28(transact); Transd.	TexMACS GMP		CD4+/CD8+ from PBMC	10^8^ total	250	TS520	Media exchanges performed without disrupting the cells	16 (cells); 20 (T cells)		Not used		CD4+ and CD8+ T cells changed from 45 and 34% to 22 and 74%.The frequency of CD4+ Tregs decreased	Transduction 60% in CD4+ and 50% in CD8+ T cells. Cells produced significant amounts of Th1 cytokine, IFN-γ and IL-2 after stimulation. Cytotoxicity ~ 60% at a 3:1 effector-to-target ratio
Zhu [197] - 2018	Used	CD3/CD28(transact); Transduct.	TexMACS GMP	3% HS	CD4+/CD8+ from PBMC	10^8^ total	NP	TS520	As medium is added or exchanged, it is programmed to shake	24.5–41.0	13	G-rex100	Signific. better	CD4+ decreased; CD8+ increased. Treg decreased. Little difference between Prodigy and G-rex in regard to phenotype	Low levels of IFN-γ. IFN-γ; all CAR-T-cell products lysed Raji cells when tested; transduction: 21 to 56.6%. better transduction in Prodigy.
Aleksandrova [203] - 2019	Not used	CD3/CD28(transact); Transd.; IL7; IL15	TexMACS GMP	HS	CD4+/CD8+ from PBMC	NR	NP	TS520	NP	46–81	12	Not used		97% T cells (range 96–99); some impurities of NKT and NK cells: median 2.9 and 0.07%, respectively. CD4/CD8 ratio of 2.4 decreased to 1.7 (range 1.1–2.3) in the final product.	Transduction: 22% on day 5 (range 17–41%) and 23% in the final product (range 21–45%).; CAR T cells were also cytotoxic against target cells
Fernández [204] - 2019	100	CD3/CD28(transact); Transd.	TexMACS GMP		CD45RA− from PBMC	1 × 10^8^ total	NP	TS520	NP	13.4–38.6	10–13	Not used		Enrichment in CD4+ vs. CD8+ T cells. Effector memory (TEM) phenotype	Cytotoxicity ≥20%. The expression of NKG2D increased during the expansion of cells. NKG2D CAR expression 55–60.6%
Marín [205] - 2019	1000	T reg beads (aCD3/aCD8); Rapamycin	TexMACS GMP	5%AB	CD4 + CD25− from PBMC	Mean: 56.9 × 10^6^	250	TS510	Shaked from day 5; media exchange was conducted. Agitation paused to facilitate bead to cell contact	Similar kinetics			G-rex	G-rex 95.6% (range 93.7–97.0; *n* = 4). Prodigy R 92.8% (range 89.8–94.5%, *n* = 4; *p* = 0.08).	Suppressive capacity varied between donors but appeared to be independent of the manufacturing regimen
NK cells															
Granzin [198] - 2015	500	Irr. EBV-LCL	TexMACS GMP	5% AB	CD56 + CD3- from PBMC	2 × 10^6^	210	TS730	Shaking after day 7	850 ± 509	14	T75	1344 ± 1135	> 99% CD3e/CD56þ,T or B cells could not be detected.	No differences in the production of IFN-γ and TNF-α and similar levels of degranulation. No reduction in telomere length. T75 and Prodigy had a comparable marker profile. NKG2C, CX3CR1 and KIR2DL2/DL3 higher in Prodigy
Oberschmidt [188] - 2019	500	IL-21, IL-15; NK MACS supplement	NK MACS	5%AB	CD56 + CD3- from PBMC	2–4 × 10^6^	250	TS310	0.17–0.3 g shaking mode was activated on day 7.	11.31–17.94	3–14	Not used		98.8–99.2%	Total NK cells expressed initially low to moderate levels of NKG2D, NKp30, NKp44, and NKp46. The surface markers increased during the 14-day expansion. Death receptors and activation markers increased. NK cell cytotoxicity increased

## Conclusions

**Fig. 3:**
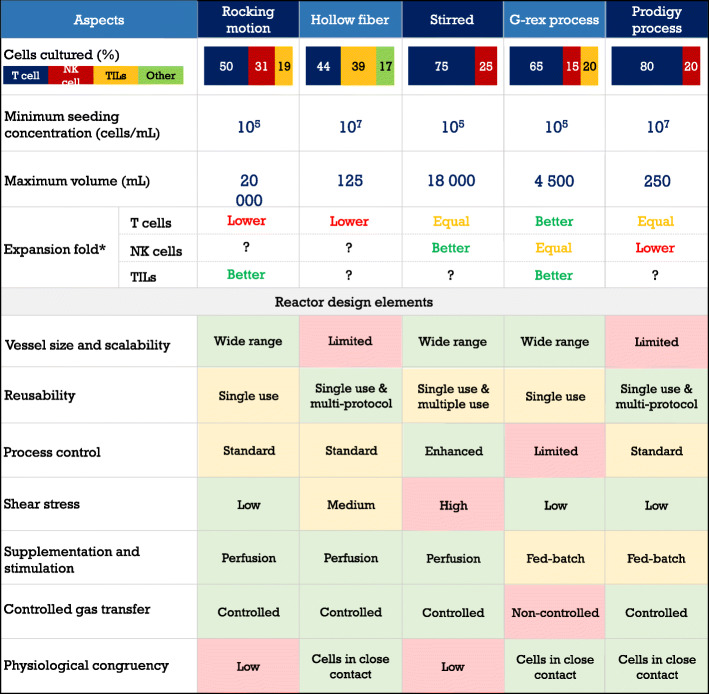
Summary of the features of the compared bioreactors. * compared to static cultures in bags or flasks

The goal of this review was to describe and compare different bioreactors that are available for lymphoid cell expansion, summarizing their design features and overall applicability to produce ACT products. Process yield, purity and product functionality were compared to overall expansion results in the context of expansion protocol diversity across different devices. The dependency on a carefully tuned stimulation strategy, high sensitivity to initial conditions and process parameters, translate into high unpredictability of the cultivation process. Reactors are flexible enough to address various ACT challenges, but also limited for some specific lymphocyte culture applications (Fig. 3) Lymphocytes are still frequently expanded totally or partly in conventional, static culture flasks or similar vessels. That is due to the fact that most immune cell types can be grown using this simple and cheap approach without special equipment, but also because those methods fulfill their purpose and, in some instances, have been shown to be even more successful than current bioreactors. The key difference between static and dynamic bioreactor cultures resides in the possibility of process intensification through quality driven approaches and the capacity of harnessing incremental knowledge from ACT manufacturing in a systematic way, compensating for source variability and cell complexity. Furthermore, the bioreactors’ ability to precisely control process parameters, mimicking in vivo conditions better than in static cultures, could be beneficial for product quality.

From the user perspective, the main challenges are found in two directions: augment ACT scale and improve process economics. That means increasing predictability of critical process stages such as stimulation schedule, feeding/splitting scheme, in-process testing or point of harvest. During the transition to phase II/III clinical trials and if cellular therapies for high impact cancers (e.g. lung and pancreatic cancer) prove to be successful, a large production scale has to be considered, needing thousands of cell therapeutic doses per year. To get a stable manufacturing pipeline for the large-scale needs, tight process control through continuous monitoring in bioreactors must be already extensively used in routine ACT expansion, which is yet to be achieved. This will be instrumental in parameter targeting for optimization, contributing towards process efficiency, increasing the accessibility of therapies to patients.

From the research perspective, the efforts in the development of ACT have been mostly product-oriented, without thoroughly considering the importance of the production process itself [14]. Bioreactors with associated computational modeling and process control will be of great benefit for understanding the mechanisms in which process parameters interact with raw material attributes and the selected stimulation strategy. In that way, cellular metabolic profiles would also provide an additional phenotypic information that can be used to guide cell fate decisions directing the expansion of preferred cell subpopulations in an automated fashion. This aspect of process automation can be used to remove sampling requirements and operator input on run conditions, thereby producing a more consistent, metabolically driven control scheme. The years to come will be framed by the transition towards a process oriented and data intensive ACT paradigm, that should translate current biological understanding into a digitally driven predictive manufacture approach, regardless of the design characteristics of the culturing platform. Every bioreactor layout will still define a specific niche within the immune cell therapeutics, but only those that able to utilize batch-to-batch process knowledge will remain competitive, as ACT is made accessible to a wider range of patients demanding higher flexibility and treatment opportunity.

## Data Availability

All data generated or analyzed during this study are included in this published article.

## Abbreviations

AB: Pooled human AB Serum
ACT: Adoptive Cell Therapy
Ag-mDCs: Autologous LNCaP-loaded mature DCs
AP: Autologous Plasma
APCs: Antigen Presenting Cells
ATMPs: Advanced Therapy Medicinal Products
BME: ß-mercaptoethanol
CAR: Chimeric Antigen Receptor
CIK: Cytokine-Induced Killer
CMV: Cytomegalovirus
CQAs: Critical Quality Attributes
CTLs: Cytotoxic T Lymphocytes
DC: Dendritic Cells
DMEM: Dulbecco’s Modified Eagle’s Medium
DO: Dissolved Oxygen
EBV: Epstein-Barr virus
EC: Extra-Capillary
FBS: Fetal Bovine Serum
GC–MS: Gas chromatography–mass spectrometry
G-CSF: Granulocyte colony-stimulating factor
GLN: Glutamine
GM-CSF: Granulocyte-macrophage Colony-stimulating Factor
GMP: Good Manufacturing Practices
HLA-DR: Human Leukocyte Antigen – DR isotype complex
HS: Human Serum
HSC: Hematopoietic Stem Cells
HSSE: Headspace Sorptive Extraction
IC: Intra-Capillary
IFN-γ: Interferon Gamma
IMDM: Iscove’s Modified Dulbecco’s Medium
Irr.: Irradiated
LAK: Lymphokine-Activated Killer
LCL: Lymphoblastoid Cell Lines
mAb: Monoclonal Antibody
NCRs: Natural Cytotoxic Receptors
NeAAs: Non-Essential Amino Acids
NK: Natural Killer
NMR: Nuclear Magnetic Resonance
OCR: Oxygen Consumption Rate
PAT: Process Analytical Technology
PBL: Peripheral Blood Lymphocytes
PBMCs: Peripheral Blood Mononuclear Cells
PCA: Principal Component Analysis
PHA: Phytohaemagglutinin
QbD: Quality by Design
REP: Rapid Expansion Protocol
RPMI: Roswell Park Memorial Institute Medium
SBSE: Stirbar Sorptive Extraction
SCGM: Stem Cell Growth Medium
TCR: T-Cell Receptor
TEFF: Effector T Cells
TILs: Tumor-Infiltrating Lymphocytes
TNFa: Tumor Necrosis Factor Alpha
Treg: Regulatory T-Cell
UCB: Umbilical Cord Blood
VCD: Viable Cell Density
VOC: Volatile Organic Compound
